# Total tanshinones ameliorates cGAS-STING-mediated inflammatory and autoimmune diseases by affecting STING-IRF3 binding

**DOI:** 10.1186/s13020-024-00980-4

**Published:** 2024-08-15

**Authors:** Chengwei Li, Jincai Wen, Xiaoyan Zhan, Wei Shi, Xiu Ye, Qing Yao, Simin Chen, Congyang Zheng, Xianlin Wang, Xinru Wen, Xiaohe Xiao, Yinghao Wang, Zhaofang Bai

**Affiliations:** 1 School of Pharmacy, Fujian University of Traditional Chinese Medicine, Fuzhou, China; 2 Department of Hepatology, the Fifth Medical Center of PLA General Hospital, Beijing, 100039 China; 3 Fifth Medical Center of Chinese, China Military Institute of Chinese Materia, PLA General Hospital, Beijing, China; 4 State Key Laboratory for Quality Ensurance and Sustainable Use of Dao-di Herbs, Beijing, 100700 People’s Republic of China

**Keywords:** Total tanshinones, Salvia miltiorrhiza, CGAS-STING, Autoimmune diseases, Acute liver injury, Inflammatory disease

## Abstract

**Background:**

An important signaling pathway connecting illness and natural immunity is the cyclic GMP-AMP synthase (cGAS)-stimulator of interferon genes (STING) pathway, but aberrant activation of this pathway is associated with the development of autoimmune and inflammatory diseases. Hence, targeted inhibition of the activation of the cGAS-STING pathway is potentially valuable in the treatment of disease. The primary active component of Salvia miltiorrhiza is total tanshinone (TTN). Research has indicated that TTN possesses noteworthy anti-inflammatory properties. However, the protective mechanism of TTN against acute liver injury (ALI) and autoimmune diseases is unknown.

**Methods:**

A model of aberrant activation of the cGAS-STING pathway was established in various cells and treated with TTN, and the expression of cGAS-STING pathway-related proteins, type I interferon, interferon stimulated genes and inflammatory factors was assessed by western blotting, real-time qPCR. Immunofluorescence analysis of the effect of TTN on the entry of associated proteins into the nucleus following aberrant activation of the cGAS-STING pathway. The effect of TTN on STING oligomerisation was investigated using 2'-3'-cyclic GMP-AMP (2',3'-cGAMP) to induce STING oligomerisation. Western blotting was used to examine the impact of TTN on the interactions of STING, tank-binding kinase 1 (TBK1), and interferon regulatory factor 3 (IRF3) after HA or Flag-labelled plasmids were transfected into HEK-293 T cells. A dimethylxanthenone-4-acetic acid (DMXAA) -induced activation model of the cGAS-STING pathway in mice was established to study the effect of TTN on aberrant activation of the cGAS-STING pathway in vivo. On the other hand, an animal model of lipopolysaccharide/D-galactosamine (LPS/D-GaIN)-induced ALI and an autoimmune disease model induced by *trex1* knockout were established to study the effects of TTN on inflammatory and autoimmune diseases mediated by the cGAS-STING pathway in vivo.

**Results:**

In several models of aberrant activation of the cGAS-STING pathway, TTN significantly inhibited the phosphorylation of STING and IRF3, thereby suppressing the expression of type I interferon, interferon-stimulated genes and inflammatory factors. Additionally, TTN prevented P65 and IRF3 from entering the nucleus after the cGAS-STING signalling pathway was abnormally activated. Subsequent research indicated that TTN was not involved in the oligomerization of STING or the integration of STING-TBK1 and TBK1-IRF3. However, TTN was found to have a substantial effect on the binding process between STING and IRF3. On the other hand, DMXAA-induced STING activation and activation of downstream signalling in vivo are inhibited by TTN. Furthermore, TTN exhibits positive treatment effects on autoimmune diseases caused by deficiency of *trex1* and LPS/D-GaIN-induced ALI.

**Conclusion:**

Our research indicates that TTN effectively treats ALI and autoimmune illnesses mediated by the cGAS-STING pathway by inhibiting the abnormal activation of this pathway.

**Graphical Abstract:**

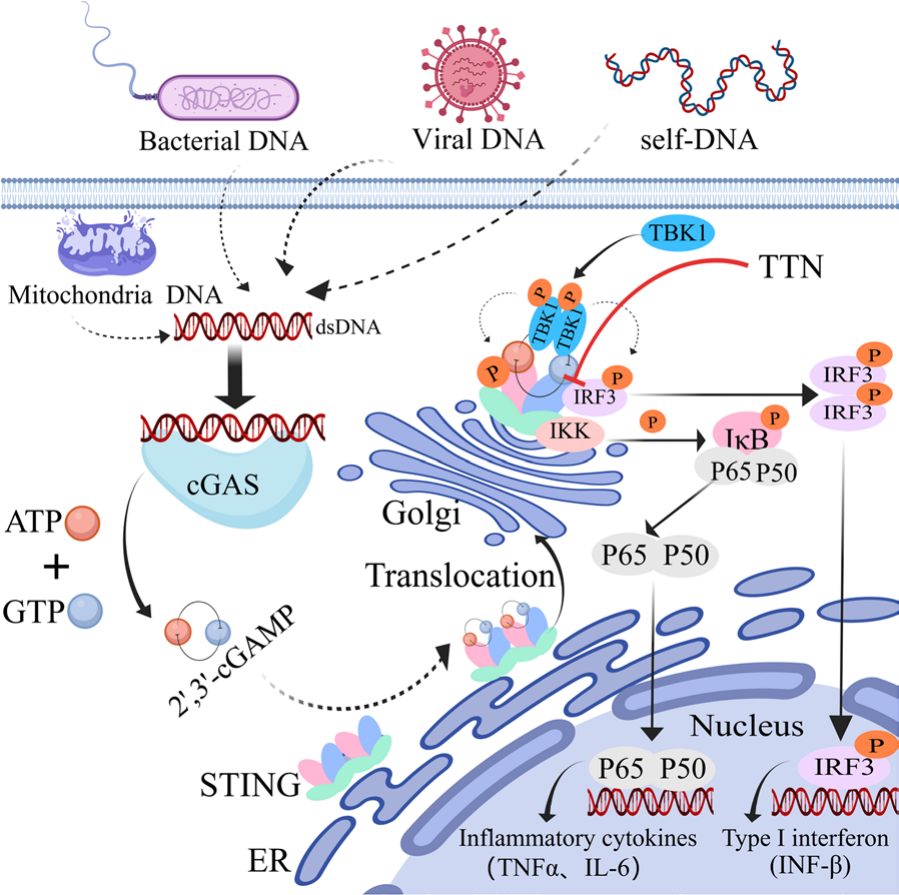

**Supplementary Information:**

The online version contains supplementary material available at 10.1186/s13020-024-00980-4.

## Introduction

Natural immunity plays a crucial part in the invasion of pathogens into the organism, which is the body’s vital barrier against microbial invasion [1, 2]. cGAS is an important abnormal state receptor that can recognize and bind to abnormal double-stranded DNA produced by pathogen infection or cellular stress, leading to the synthesis of 2',3'-cGAMP [3, 4]. 2',3'-cGAMP transmits signals to the STING dimer, which induces oligomerization of STING and translocation into Golgi vesicle [5]. Activated STING recruits and phosphorylates TBK1, and phosphorylated TBK1 in turn phosphorylates STING. The complex formed by STING and TBK1 recruits and dimerizes IRF3, and the dimerized IRF3 translocate to the nucleus [6, 7]. Upon activation, STING also activates nuclear factor-kappa B (NF-κB) signaling, resulting in the migration of the P65/P50 dimer into the cell’s nucleus to work together with IRF3 to co-regulate the type I interferon and inflammation-related gene expression levels [8, 9].

Activation of cGAS-STING triggers the immune response, it could enhance the ability of immune cells to kill antigens through multiple pathways and defend against invasion of pathogens [10, 11]. However, abnormalities in the structure or function of this pathway can also result in the occurrence of Autoimmune and inflammatory diseases, Examples include systemic lupus erythematosus (SLE), nonalcoholic fatty liver disease (NAFLD), and Aicardi-Goutières syndrome (AGS). According to reports, prolonged stimulation of aberrant DNA causes the cGAS-STING pathway to be activated aberrantly. This, in turn, causes the continuous synthesis and release of type I interferons and inflammatory cytokines, which are important in the development of inflammatory or autoimmune diseases [12]. *Trex1* is a cytoplasmic non-restriction exonuclease that efficiently hydrolyzes excess DNA fragments in the cytoplasm. When an organism’s *trex1* gene is deleted, abnormal DNA accumulates in the cytoplasm. When cGAS identifies cytoplasmic DNA, it triggers downstream signalling pathways that cause type I interferons and inflammatory cytokines to be released continuously, which is an essential cause of autoimmune illness development [13]. ALI has been linked to the cGAS-STING pathway, according to previous research. In the event of ALI, cell death, viral infection, or other events that may cause DNA damage may occur [14, 15]. Inflammatory and immunological reactions in the body can result from these circumstances because they can cause the release of mitochondrial DNA into the cytoplasm, which subsequently binds to the DNA receptor cGAS, activating the cGAS-signaling pathway and boosting the type I IFN response. Consequently, the cGAS-STING pathway has come to be recognized as a crucial pathway in the control of inflammation in relation to infection, cellular stress, and tissue damage. In summary, in many human inflammatory illnesses, the development of inhibitors targeting the cGAS-STING signaling axis is significant.

Chinese medicine is particularly unique in the treatment and prevention of diseases [16], where the discovery of new potential lead compounds from the natural products of Chinese medicine is an important direction in the treatment of diseases [17]. Salvia miltiorrhiza is a very widely used traditional Chinese medicine (TCM) with excellent therapeutic effects on cardiovascular diseases, oxidative stress, and tumors, and inflammation. TTN is an important constituent of Salvia miltiorrhiza. The main components of TTN include compounds such as Dihydratanshinone I, Tanshinone I, Cryptotanshinone and Tanshinone IIA, etc. [18, 19], which have some anti-inflammatory as well as anticancer effects inside and outside the carrier. At the same time, TTN is also the main component of the clinical drug Tanshinone Capsules. Tanshinone Capsules has a broad-spectrum antimicrobial effect, but the study of its exact mechanism of action is still limited [20, 21]. Evaluating the anti-inflammatory effects of TTN will help to better elucidate its drug properties and promote the modernization of TCM, as well as enable the drug repurposing of Tanshinone Capsules for the treatment of more characteristic diseases.

In this research, we revealed that TTN specifically inhibited cGAS-STING pathway activation. Mechanistically, TTN could block STING-IRF3 binding but had no effect on STING oligomerization or STING-TBK1 or TBK1-IRF3 binding. Importantly, in vivo experiments, TTN dramatically inhibited the cGAS-STING pathway's activation caused by DMXAA. Moreover, it also had a good therapeutic effect on *trex1*^*−/−*^ induced autoimmune diseases and LPS/D-GalN-induced ALI. Hence, We think that TTN could be a modulator for the management of inflammatory or autoimmune diseases that are caused by the cGAS-SITNG pathway.

## Materials and methods

### Animals

We purchased seven-week-old C57BL/6 J wild-type (WT) mice from SPF Biotechnology Co., Ltd. (Beijing, China). The *trex1* mice were donated by the Jackson Laboratory (Genetics Research, Bar Harbor, Maine, USA). Heterozygous male and female *trex1*^*−/−*^ mice were further mated to produce offspring. Mouse genotypes were detected using standard PCR methods. All animals were housed in an environment free of specific viruses and pathogens, maintained in twelve hours of light and twelve hours of darkness. The Animal Ethics Committee of the Fifth Medical Center of the General Hospital of the People’s Liberation Army (PLA) granted licenses for all phases of animal testing.

### ***Trex1***^***−/−***^induced autoimmune diseases

We administered excipients and TTN (200 mg/kg) intragastrically to WT and *trex1*^*−/−*^ mice every day. Mice in the WT + TTN group were also gavaged with TTN (200 mg/kg). 14 days later, the mice were killed, and the heart, liver, kidney, and lung tissues were collected. We observed pathological changes in the tissue using hematoxylin–eosin staining (H&E). The mRNA levels of IFN-β, IL6, CXCL10, and ISG15 in the heart, liver, kidney, and lung tissues were assessed using qPCR.

### DMXAA-induced activation of the cGAS-STING pathway in vivo

After 7 days of domestication, C57BL/6 mice were randomly divided into 4 groups and gavaged with the corresponding vehicles or TTN (100 or 200 mg/kg) for 7 consecutive days in a row, respectively. One hour after the final treatment, both the treated and model groups received an intraperitoneal injection of DMXAA (25 mg/kg) [22]. Four hours later, collect the mouse serum, and the abdominal cavities of the mice were rinsed using PBS to access the intraperitoneal lavage fluid. Lastly, ELISA kits were used to identify TNF-α, IL-6, and IFN-β expression in blood and peritoneal lavage fluid.

### Acute liver injury model

After being domesticated and housed for 7 days, male C57BL/6 mice were split into six groups at random. The treatment group received constant gavage for 7 days, while the other groups received equal doses of excipients. The mice in the H151 group received an intraperitoneal injection of H151 (10 mg/kg) at the same time as the last dosage. Mice in all groups but the control and TTN (200 mg/kg) groups received an intraperitoneal injection of LPS/D-GaIN 1 h following the final day of treatment. Serum and liver tissues were extracted from the animals four hours later. Type I interferon and inflammatory factor expression levels in serum were detected by ELISA kit [23]. The degree of liver tissue damage was assessed using H&E staining. Transcript levels of type I interferon and inflammatory cytokines were measured using a qPCR assay.

### Quality control of total tanshinones

An Agilent 100-5-C18 4.6*250 mm plus column was used for the chromatographic study, with methanol serving as mobile phase A and a 0.1% phosphoric acid solution serving as mobile phase B. The column temperature was 30 °C, the flow rate was 0.8 ml/min, and the detection wavelength was 270 nm. In Supplementary Fig. 2, the UHPLC chromatogram of TTN is displayed. Through a comparison of the chromatographic peak retention periods with established chemical standards, it was determined that the predominant constituents of TTN were Dihydratanshinone I, Tanshinone I, Cryptotanshinone and Tanshinone IIA.

### Cell viability assay

In 96-well plates, BMDMs or THP-1 were cultured, and after cell adhesion, they received exposure to various TTN concentrations. After twelve hours hours, the CCK8 reagent was applied to the well plates. The plates were placed in an incubator and left for 1 h. By measuring the optical density at 450 nm with enzyme labeling equipment, the impact of TTN on cell survival was evaluated.

### Reagents and antibodies

TTN (XS-20220526-21) and DMXAA (DM0234) were purchased from Chengdu DeSiTe Biological Technology Co, Ltd (SiChuan, China), Poly (I:C) and DMSO are from Sigma. diABZI (HY-112921A) and Cell Counting Kit-8 (HY-K0301) are obtained from MedChemExpress. 2',3'-cGAMP (B8632) was from APEBIO. Mouse TNF-α ELISA kits (1217202) and mouse IL-6 (1210602) ELISA kits are purchased from Dakewe. Mouse IFN-β ELISA kit is from Invivogen. AST kit (C010-2-1) and ALT kit (C009-2-1) were purchased from Nanjing Jiancheng Biotechnology Co., LTD. DMEM (CM10013), penicillin–streptomycin (CC004) and RPMI-1640 (CM10040) were purchased from Macgene. Anti mouse p-IRF3 (GTX86691) was bought from GeneTex, Anti-IRF3 (11312–1-AP), anti-STING (19851–1-AP), anti-HA tag (51064-2-AP), anti- DDDDK tag (80010-1-RR) anti-HSP90 (13171–1-AP) was obtained from Proteintech. Anti-Human p-IRF3 (ab76493) was from Abcam.

### Cell culture

For 5 days, mouse femur-derived bone marrow macrophages (BMDMs) were cultivated in DMEM with 1% penicillin–streptomycin (P/S) and 10% fetal bovine serum (FBS) added. While adding 100 μg/mL of macrophage colony-stimulating factor (MCSF) to induce differentiation. HEK-293 T and HEK-293 were similarly cultivated in DMEM and processed for passaging every other day. Tohoku Hospital Pediatrics-1 (THP-1) was raised on RPIM 1640 medium with 1% P/S and 10% FBS. The medium was replenished every other day, and when seeding plates, PMA was applied to encourage wall adhesion and differentiation.

### cGAS-STING signaling pathway activation

We seeded BMDMs and THP-1 at densities of 1.1 × 10^6^/mL and 1.5 × 10^6^/mL, respectively, in 24 seeded plates. After fifteen hours, cells were exposed to opti-mem containing TTN and transfected with ISD (an immune-stimulatory DNA, the primer sequences used are shown in Table 1), 2',3'-cGAMP, DMXAA and diABZI (Several agonists of STING) after one hour. After two hours, cells were lysed with 1 × loading, and whole-cell lysates were used for immunoblotting [24]. Following four hours of stimulation, Trizol was used to lyse the samples, and RNA was then isolated from the cells for qPCR.

**Table 1:** Primer sequence

Target gene	Sequence (5′-3′)
Mouse Actin	GGCTGTATTCCCCTCCATCG
	CCAGTTGGTAACAATGCCATGT
Mouse IFN-β	TCCGAGCAGAGATCTTCAGGAA
	TGCAACCACCACTCATTCTGAG
Mouse CXCL10	ATCATCCCTGCGAGCCTATCCT
	GACCTTTTTTGGCTAAACGCTTTC
Mouse ISG15	GGTGTCCGTGACTAACTCCAT
	CTGTACCACTAGCATCACTGTG
Mouse TNF-α	GGGCAGTTAGGCATGGGAT
	TGAGCCTTTTAGGCTTCCCAG
Mouse IL-6	CACTTCACAAGTCGGAGGCT
	CTGCAAGTGCATCATCGTTGT
Human Actin	CATGTACGTTGCTATCCAGGC
	CTCCTTAATGTCACGCACGAT
Human IFN-β	TCCAAATTGCTCTCCTGTTG
	GCAGTATTCAAGCCTCCCAT
Human CXCL10	TGGCATTCAAGGAGTACCTC
	TTGTAGCAATGATCTCAACACG
Human TNF-α	CCTCTCTCTAATCAGCCCTCTG
	GAGGACCTGGGAGTAGATGAG
Human IL-6	ACTCACCTCTTCAGAACGAATTG
	CCATCTTTGGAAGGTTCAGGTTG
ISD	TACAGATCTACTAGTGATCTATGACTGATCTGTACATGATCTACA
	TGTAGATCATGTACAGATCAGTCATAGATCACTAGTAGATCTGTA

### ELISA

As required by the manufacturer, assays for IFN-β, IL-6, and TNF-α in serum and peritoneal lavage fluid [25].

### Immunofluorescence

BMDMs or THP-1 were seeded in 96-well plates and treated with TTN for one hour after fifteen hours, followed by treatment with diABZI for two hours. The plates underwent a PBS wash, 4% paraformaldehyde fixation, and a 15-min Triton X-100 permeabilization process. The cells were then left at room temperature for an additional 1 h and then incubated overnight with antibodies against P65 or IRF3. Hoechst stained the nuclei blue.

### Immunoprecipitation study

After transfecting the HA and FLAG-labelled plasmids into HEK-293 T cells for eighteen hours, the cells were exposed to TTN for an additional six hours. Cells were lysed using a lysate containing a 1% protease inhibitor. Cell suspension was collected by centrifugation at 12,800 g for 10 min at 4 °C. 1/10 of the cell supernatant was added to 5 × loading as input, and the rest of the supernatant was incubated with an anti-Flag M2 affinity gel pellet for immunoprecipitation by co-incubation at 4 °C for 4 h. After that, the immunoprecipitates were centrifuged, 1 × loading was added as IP, and finally the level of the target proteins was measured by immunoblotting [26, 27].

### Overexpression experiment

Twenty-four well plates were seeded with 5.0 × 10^5^ HEK-293 cells. Following cell adhesion, the cells were subjected to DMSO or TTN treatment after being transfected for eighteen hours with the tag proteins Flag-STING, Flag-TBK1, and Flag-IRF3. Samples were taken for qPCR or western blot six hours later.

### Total RNA extraction and reverse transcription

Cell samples or tissue samples were lysed with Trizol, and chloroform was added to mix the cells. After the cells were centrifuged at 4 °C for 15 min at 12,000 g,The upper aqueous phase was collected and extracted with the same volume of isopropanol, and the extract was allowed to stand for 10 min before being centrifuged at 4 °C for 8 min at 12,000 g. At this point a total RNA pellet could be seen at the bottom of the tube, the supernatant was discarded, and after adding an equivalent volume of 75% ethanol, the whole RNA was centrifuged at 7000 g for three minutes at 4 °C to wash it away. The supernatant was discarded, excess ethanol was evaporated at room temperature and resuspended with 30 ul of sterile, enzyme-free water. Finally, reverse transcription was performed following the manufacturer's guidelines to obtain cDNA [28].

### Quantitative real-time PCR

Real-time quantitative PCR of cDNA was conducted using SYBR Green qPCR Mix, and the expression of target genes was detected using the ΔΔC_T_ method. In this experiment, β-Actin were used as internal references. Table 1 displays the primer sequences that were used in this investigation.

### Western blots

Methods for protein immunoblotting have been previously described [29].

### STING oligomerization assay

After putting BMDMs in 12-well plates and leaving them there for fifteen hours, the cells were exposed to opti-mem containing TTN for 1 h, followed by initiation of the cGAS-STING pathway after transfection with 2',3'-cGAMP. Two hours later, cells were lysed with lysis buffer, and the cell suspension was collected by centrifugation at 4 °C for 10 min at 8000 g. The cell supernatant was collected, and 1/5 was added to 1 × loading containing SDS as a natural denaturing sample. To the remaining supernatant, add 5 × loading without SDS as a non-denatured sample. The above samples were loaded into natural gels with or without SDS and finally tested for effects on STING protein expression [30].

### Statistical analysis assay

Prism software was used to perform the required statistical analyses of qPCR experiment results and ELISA kit results. All experimental notation results are exhibited as mean ± SEM; for statistical analyses between two groups, an unpaired Student's t-test was used; and for statistical analyses between multiple groups, a one-way ANOVA followed by Dunnett’s post hoc test was used. P < 0.05 was considered statistically significant.

## Results

### TTN inhibits cGAS-STING pathway activation

**Fig. 1:**
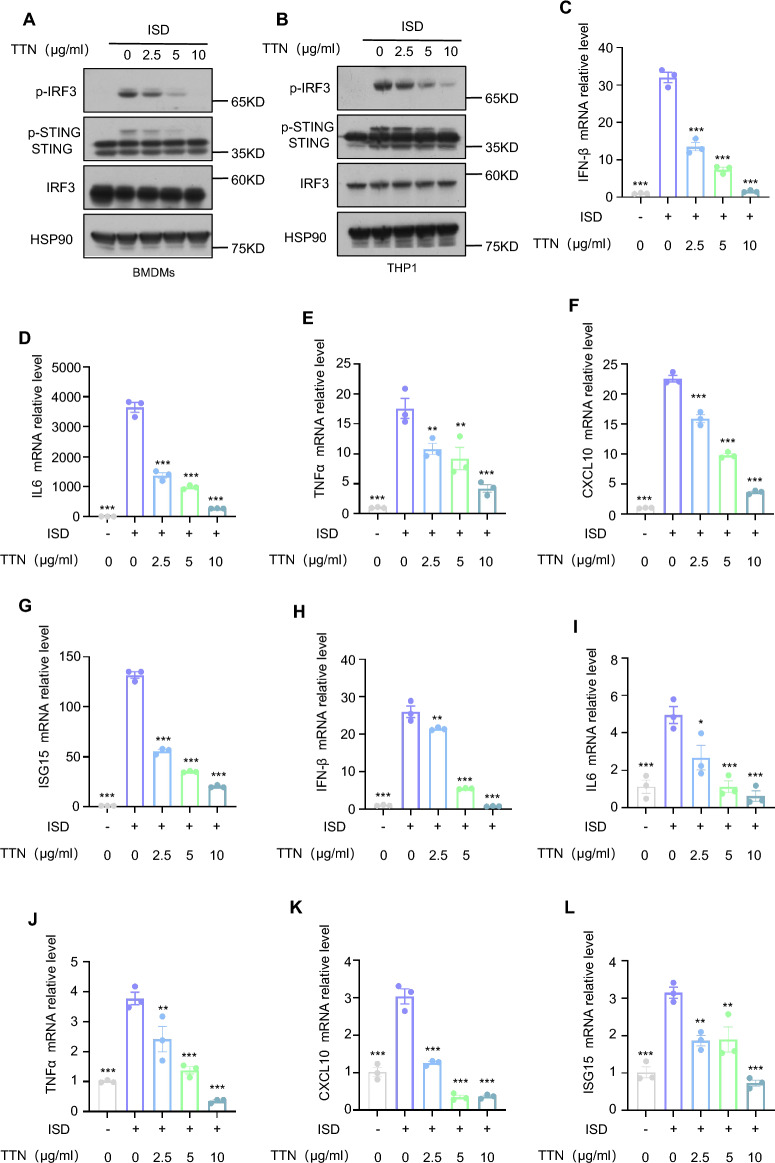
TTN inhibits activation of the cGAS-STING pathway. **A**, **B** After one hour of opti-mem pretreatment with or without TTN (2.5, 5, 10 μg/ml) for BMDMs or PMA-primed THP-1, two hours were spent treating them with ISD (3 μg/ml), and the effect of TTN on the relevant proteins was detected with the indicated antibodies. **C**–**L** BMDMs or PMA-primed THP-1 were pretreated with opti-mem with or without TTN for 1 h and then treated with ISD for four hours. The effect of TTN on IFN-β, IL-6, TNF-α, CXCL10 and ISG15 mRNA was detected by qPCR assay. Data in (**C**–**L**) are presented as Mean ± SEM for the three biological samples, one-way ANOVA followed by Dunnett’s post hoc test was used to detect statistical differences between the analyzed multiple groups. *p < 0.05, **p < 0.01 and ***p < 0.001 vs. the stimulated group, NS, not significant

To investigate the effect of TTN on the cGAS-STING pathway, we first examined the effect of TTN on the cell viability of BMDMs. The results indicated that TTN below 20 μg/ml had no influence on cell viability (Supplementary Fig. 1A). One hour after the administration of TTN, we established the model of cGAS-STING pathway activation by transfecting ISD in BMDMs, and assessed the effect of TTN by detecting the expression levels of relevant proteins downstream of the pathway. The results illustrated that TTN could greatly inhibit the level of phosphorylated IRF3 and STING induced by ISD stimulation, while there was no impact on IRF3 and STING expression (Fig. 1A). Furthermore, we also examined the effect of TTN on the mRNA level of type I interferon-related genes (IFN-β, ISG15, CXCL10) and inflammatory factors (IL6,TNFα) downstream of the pathway using a qPCR assay. As with the immunoblotting results, TTN exhibited significant inhibitory effects on downstream related genes (Fig. 1C–G).

The pharmacological activity of the compounds produces different effects in different species and in different types of cells. THP-1 is a human leukemia monocyte line that has been widely used to investigate monocyte/macrophage function, mechanisms, signaling pathways, and nutrient and drug transport. To further validate the effect of TTN on the cGAS-STING signaling pathway, we performed the same experiment on THP-1. A cytotoxicity assay suggested that TTN did not exhibit cytotoxicity at less than 20 μg/ml in THP-1 (Supplementary Fig. 1B). Second, immunoblotting and qPCR results also showed that TTN significantly inhibited ISD-induced aberrant activation of the cGAS-STING pathway in THP1 (Fig. 1B, H–L).

### TTN specifically inhibits activation of the cGAS-STING pathway under multiple stimuli

**Fig. 2:**
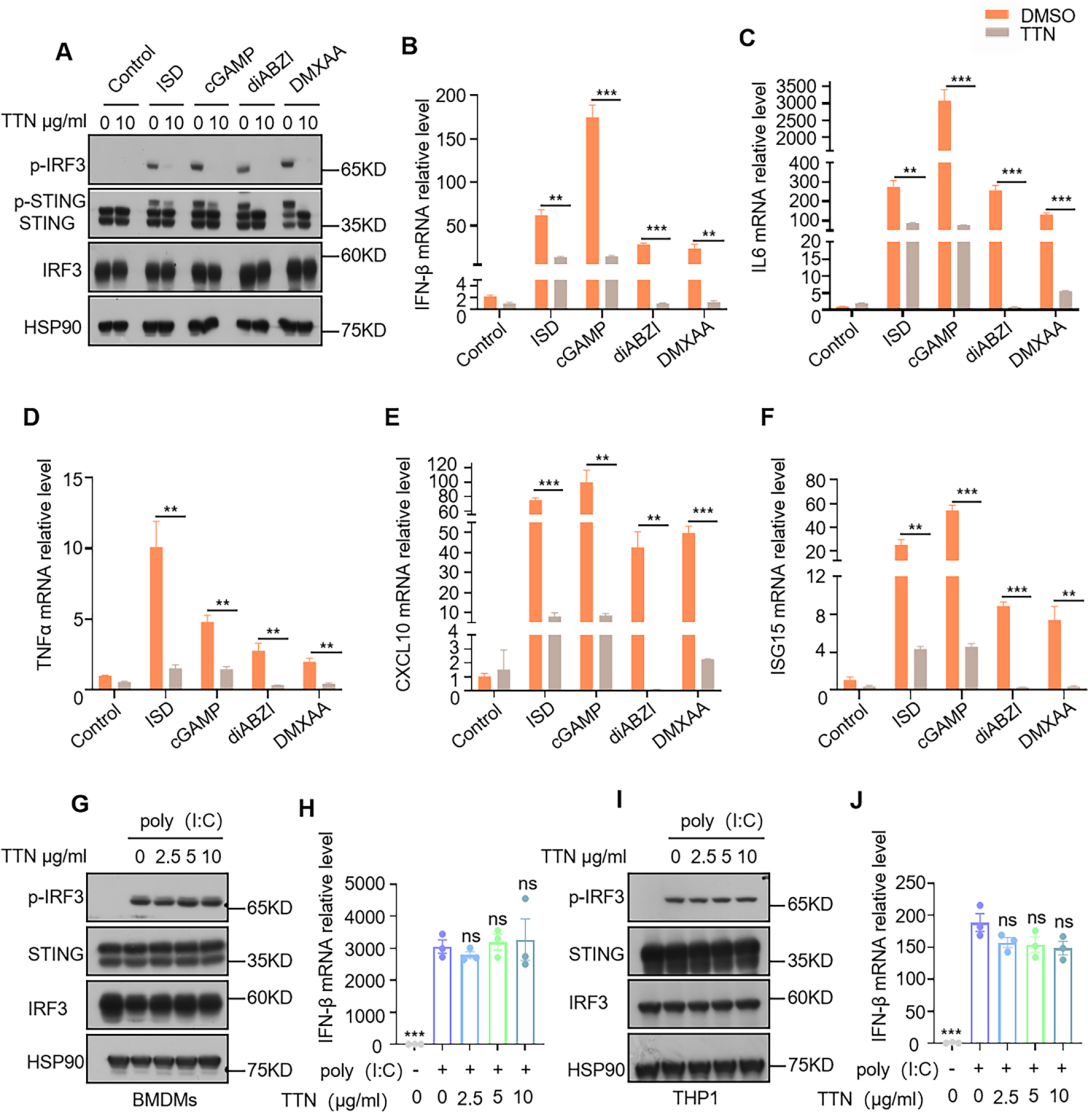
TTN specifically inhibits multiple agonist-induced activation of the cGAS-STING pathway. **A** After one hour of treatment with either DMSO or TTN (10 μg/ml), BMDMs were stimulated for two hours with ISD (3 μg/mL), 2',3'-cGAMP (2 μg/mL), diABZI (12.5 μg/mL), or DMXAA (10 μmol/L). The effect of TTN on the relevant proteins was detected with the indicated antibodies. **B**–**F** After one hour of treatment with DMSO or TTN (10 μg/mL), BMDMs were stimulated for 4 h with ISD, 2',3'-cGAMP, diABZI, or DMXAA. The qPCR technique was utilized to assess the expression levels of IFN-β, IL-6, TNF-α, CXCL10 and ISG15 mRNA. **G**–**J** BMDMs or PMA-pretreated THP-1 was treated with DMSO or different concentrations of TTN for one hour. Cells were stimulated by transfection given with poly(I:C) (2 μg/mL) for 2 h. Cell lysates were collected and assayed for the expression of p-IRF3, IRF3 and STING by immunoblotting.Stimulated for four hours, Samples for qPCR were used to extract their RNA and detect the reverse transcription level of IFN-β. Data in (**B**–**F**) are presented as Mean ± SEM for the three biological samples, unpaired Student’s t-test was used to detect statistical differences between two groups. *p < 0.05, **p < 0.01 and ***p < 0.001 vs. the stimulated group, NS, not significant

The cGAS-STING pathway can be activated by a variety of STING agonists, including 2', 3'-cGAMP, diABZI, and DMXAA. To further validate the inhibitory effect of TTN, we established a model of aberrant activation of the cGAS-STING pathway induced by a variety of stimuli in BMDM cells to assess the potency of TTN. It was shown that TTN is an inhibitor of the classical cGAS-STING signaling pathway and has favorable inhibitory effect on both phosphorylated IRF3 and phosphorylated STING induced by various agonists (Fig. 2A). Additionally, we used qPCR to investigate TTN's impact on the mRNA expression levels of type I interferons, interferon-stimulated genes, and inflammatory factors downstream of the cGAS-STING pathway in the presence of various agonists. The findings demonstrated that TTN efficiently decreased the mRNA levels of genes related to type I interferon and inflammatory factors that these agonists generated (Fig. 2B–F). The cGAS-STING pathway senses aberrant DNA inside and outside the cell and stimulates the body to initiate an immune response, while some pathogens also produce RNA. Synthetic double-stranded RNA called Poly (I:C) triggers the RIG-1-MAVS signaling pathway, which in turn produces type I interferon [31]. We examined the effect of TTN on the RIG-1-MAVS pathway under Poly (I:C) stimulation in BMDMs and THP-1 and showed that TTN had no effect on the phosphorylation of IRF3 and the expression of type I interferon (Fig. 2G–J), suggesting that TTN is specific for inhibiting aberrant DNA-induced activation of the cGAS-STING pathway. These results indicate that TTN specifically inhibited the activation of the cGAS-STING signaling pathway induced by aberrant DNA in BMDMs, but did not inhibit the RIG-1-MAVS signaling pathway induced by aberrant RNA.

### TTN inhibits diABZI-driven nuclear translocation of P65 and IRF3

**Fig. 3:**
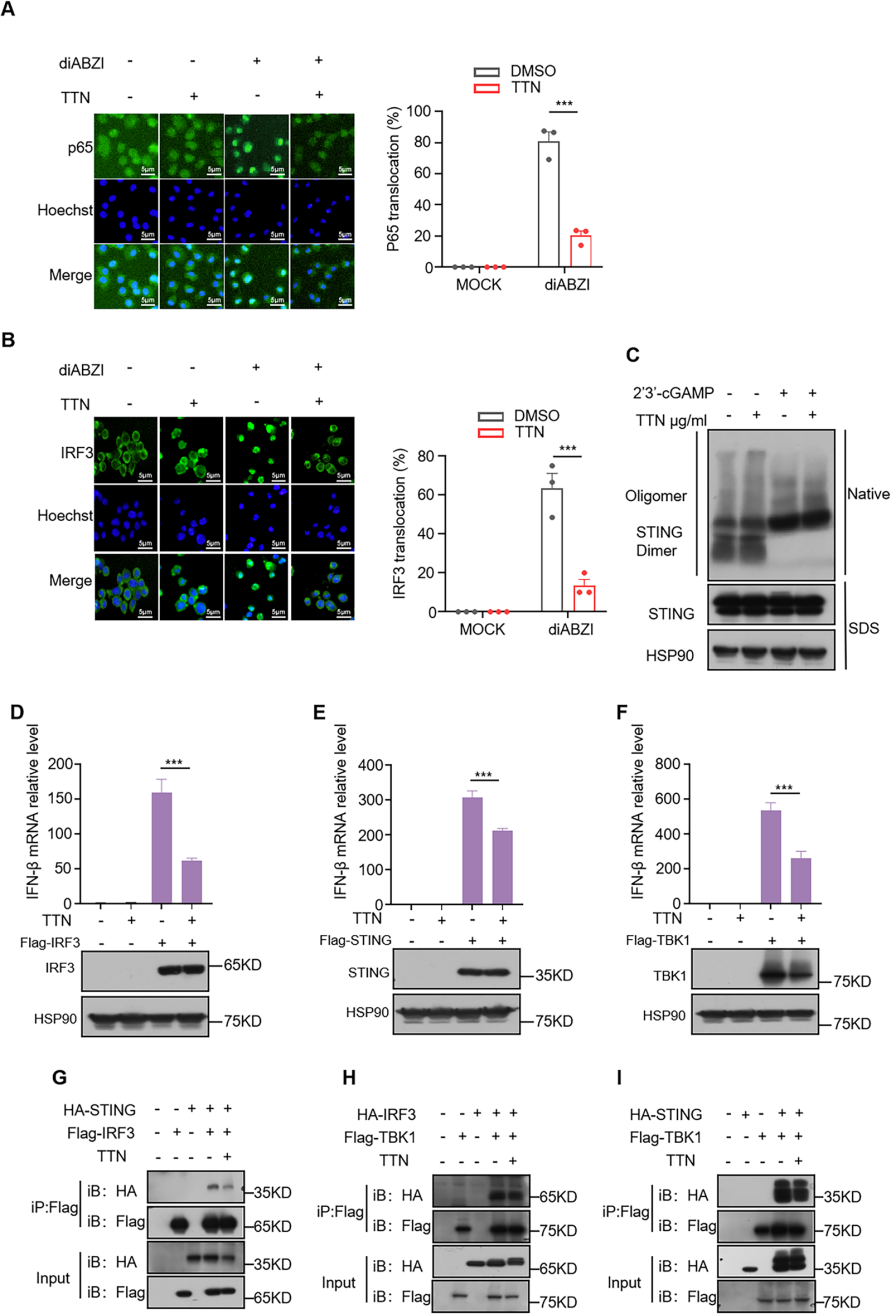
TTN affects the binding between SITNG and IRF3. **A**, **B** BMDMs (**A**) or THP-1 (**B**) were treated with DMSO or TTN (10 μg/mL) for one hour, and then stimulated with diABZI for two hours and stained for P65 (**A**) and IRF3 (**B**). At least three different images were taken in each sample (scale bar: 5 μm). **C** BMDMs were treated with TTN (10 μg/mL) for one hour and then with 2',3'-cGAMP for 2 h. Immunoblotting was performed to detect oligomerization of STING and STING protein expression levels. **D**–**F** HEK-293 cells were transfected with Flag-IRF3, Flag-STING, or Flag-TBK1 for sixteen to eighteen hours, and after that, they were exposed to TTN (10 μg/mL) for six hours. After obtaining whole cell lysates, the designated antibody was used to perform an immunoblot. The qPCR was used to identify samples for the detection of IFN-β mRNA. **G**–**I** After transfecting HA- and Flag-tagged plasmids into HEK-293 T cells for sixteen to eighteen hours, the cells were treated with or without TTN (10 μg/mL), immunoprecipitated using anti-Flag M2 affinity gel beads, and immunoblotting was used to detect protein–protein interactions. Data in (**D**–**F**) are presented as Mean ± SEM for the three biological samples, one-way ANOVA followed by Dunnett’s post hoc test was used to detect statistical differences between the analyzed multiple groups. *p < 0.05, **p < 0.01 and ***p < 0.001 vs. the stimulated group, NS, not significant

The nuclear translocation of P65 and IRF3 is a crucial step in the activation of the cGAS-STING pathway, which is essential for the subsequent expression of type I interferon genes and pro-inflammatory cytokine genes [32]. We first examined the effect of TTN on P65 nuclear translocation using immunofluorescence. P65 gradually translocated into the nucleus following diABZI activation of the cGAS-STING pathway, and TTN was able to inhibit this tendency (Fig. 3A). We also saw that when diABZI activates this pathway, the nuclear translocation of IRF3 can be inhibited by TTN (Fig. 3B). In conclusion, TTN inhibited the nuclear translocation of IRF3 and P65 under diABZI stimulation.

### TTN inhibits the cGAS-STING pathway by affecting STING-IRF3 interaction

It was reported that STING oligomerization is a major upstream event during cGAS-STING pathway activation, which has a direct regulatory influence on the next transfer of STING and the formation of STING signalosomes. We next verified the effect of TTN on STING oligomerization and found that TTN did not influence STING oligomerization in the presence of 2',3'-cGAMP stimulation (Fig. 3C), suggesting that TTN may influence the formation of pathways downstream of the cGAS-STING pathway. Next, to learn more about the proteins that TTN might target, we put foreign plasmids into HEK-293 cells and used qPCR to measure the level of IFN-β mRNA expression. We discovered that the expression level of IFN-β was elevated after transfection with Flag-STING, Flag-TBK1 or Flag-IRF3, suggesting that the cGAS-STING pathway is also activated after overexpression of the relevant proteins. We found that TTN could significantly decrease the expression level of IFN-β induced by Flag-STING, Flag-TBK1 or Flag-IRF3 (Fig. 3D–F), and therefore we hypothesized that TTN could have affected the formation of the STING signalosome. We investigated the impact of TTN on the cGAS-STING pathway by transfecting exogenous HA- and Flag-tagged plasmids on HEK-293 T cells and pulling them down with anti-M2 Flag agarose beads in order to further validate the exact mechanism of action of TTN. TTN was found to significantly inhibit STING-IRF3 binding but had no effect on STING-TBK1 or IRF3-TBK1 binding (Fig. 3G–I). This suggests that TTN inhibits the activation of cGAS-STING pathway by influencing STING-IRF3 binding.

### TTN inhibits DMXAA-induced activation of STING and downstream signaling in vivo

**Fig. 4:**
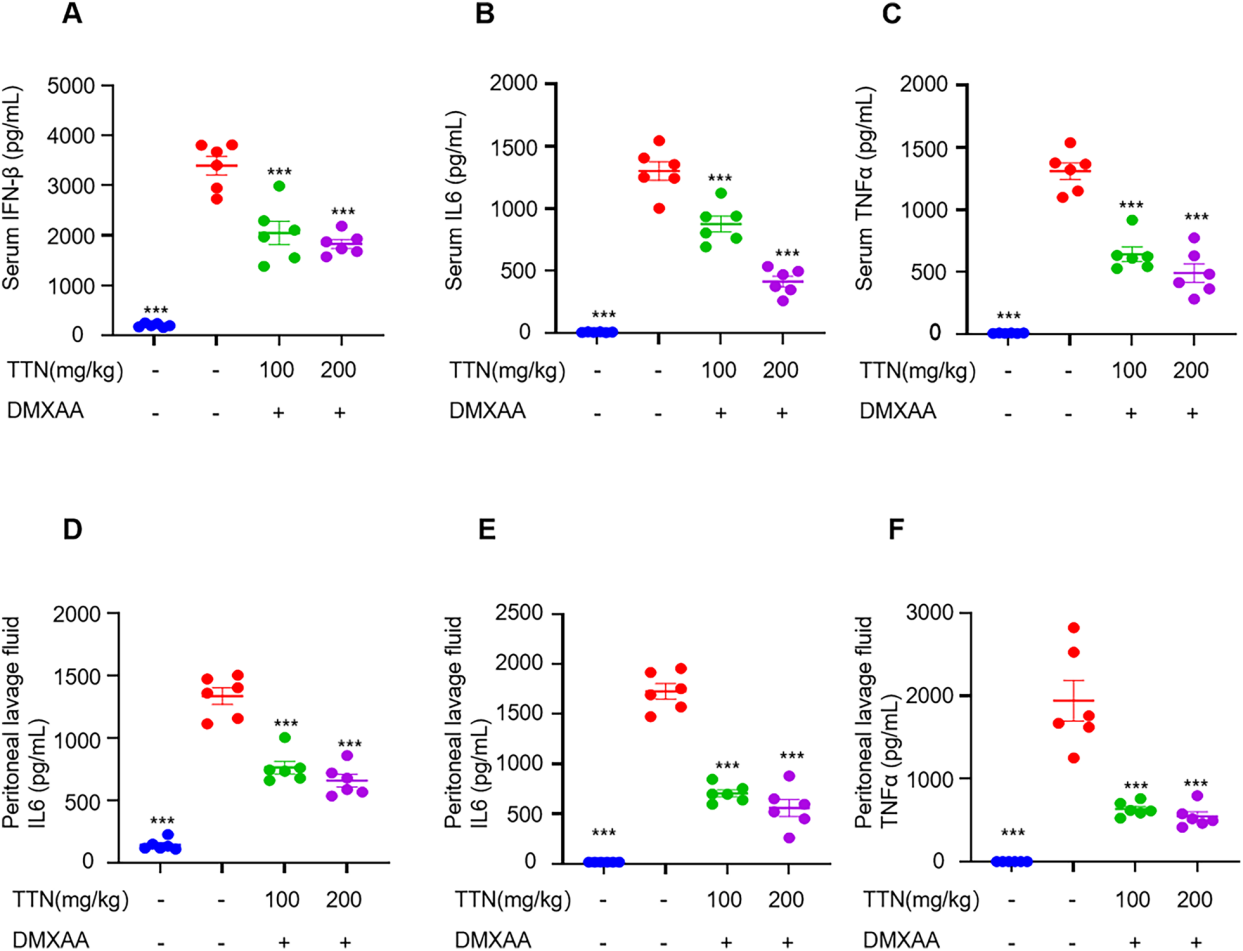
TTN inhibits DMXAA-induced activation of STING downstream signalling in vivo. **A**–**F** C57BL/6 J mice were randomly divided into four groups (n = 6 per group). The administration group received the appropriate dose of tanshinone by gavage for 7 consecutive days, while the blank and DMXAA groups received equal amounts of excipients, and one hour after the last administration, the mice received an intraperitoneal injection of DMXAA (25 mg/kg), except for the blank group. Serum and peritoneal lavage fluid were taken after four hours. The levels of IFN-β, TNF-α and IL-6 in serum and peritoneal lavage fluid were determined by enzyme-linked immunosorbent assay (n = 6 per group). Data in (**A**–**F**) are presented as Mean ± SEM, one-way ANOVA followed by Dunnett’s post hoc test was used to detect statistical differences between the analyzed multiple groups. *p < 0.05, **p < 0.01 and ***p < 0.001 vs. the model group, NS, not significant

A new STING agonist called DMXAA was shown to work in mice to activate STING and the signaling pathways that follow. We injected mice with DMXAA to activate STING in vivo and administered different concentrations of TTN, and examined the therapeutic effects of TTN by detecting the level of interferon and inflammatory factors in the serum and peritoneal lavage fluid of mice [33]. Results indicated that the expression of type I interferon and inflammatory factors was elevated in vivo in mice stimulated by DMXAA. The expression levels of IFN-β, IL6, and TNFα in the serum of mice were significantly reduced after TTN treatment, suggesting that TTN can effectively inhibit the activation of the cGAS-STING pathway induced by DMXAA in vivo (Fig. 4A–F).

### TTN attenuates autoimmune responses caused by *trex1* mutation

**Fig. 5:**
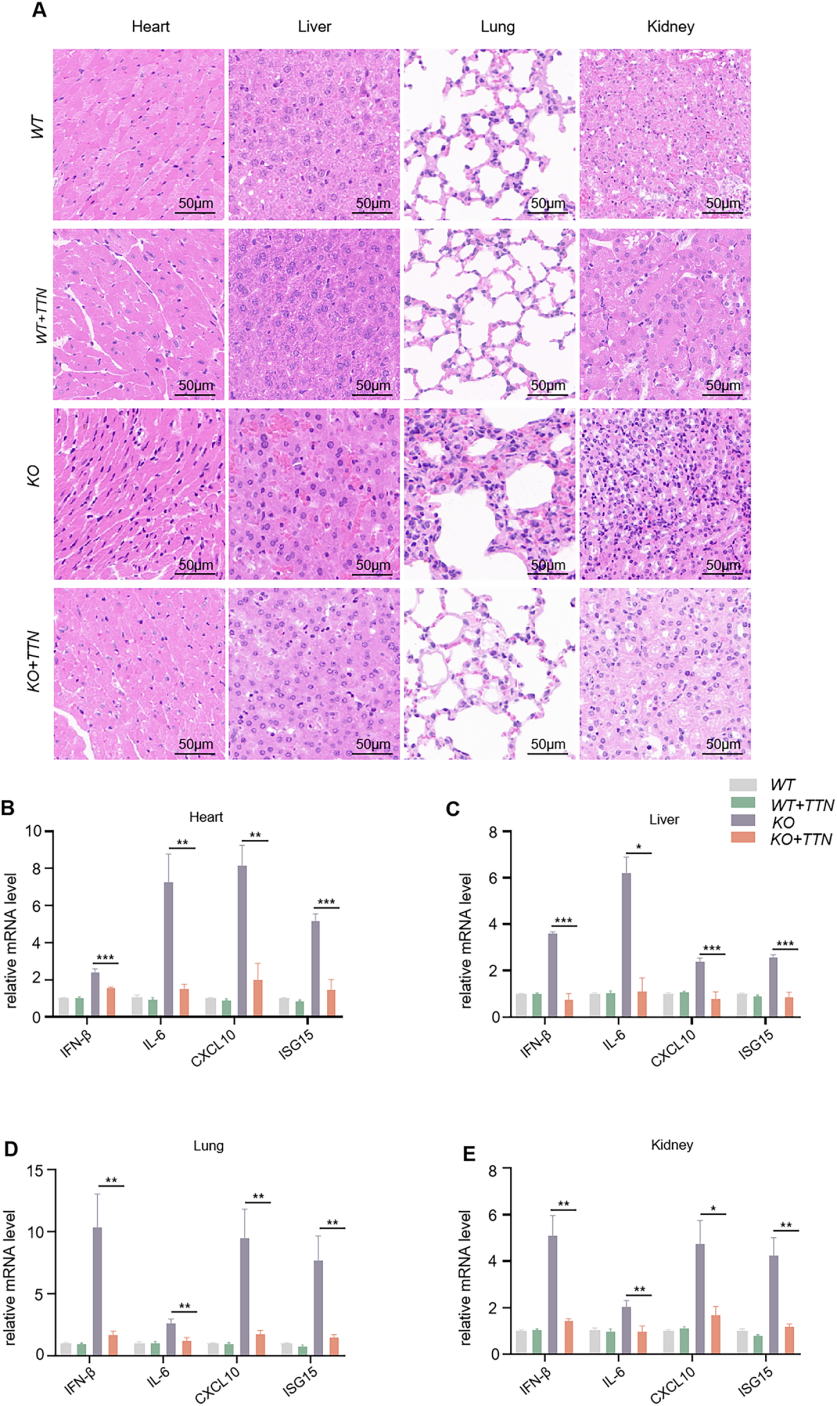
TTN attenuates autoimmune responses in *trex1-*deficient mice. **A** Representative images of H&E staining in in heart, liver, lung and kidney tissues (n = 6 per group). **B**–**E** WT or *trex1*^*−/−*^ mice gavaged with TTN (200 mg/kg) or excipients (n = 6 per group) for a period of fourteen days. Then, tissues from the heart (**B**), liver (**C**), lung (**D**), and kidney (**E**) were gathered, and qPCR was used to ascertain the mRNA expression of the indicated genes. Data in (**B**–**E**) are presented as Mean ± SEM for the 6 mice per group, unpaired Student's t-test was used to detect statistical differences between two groups. *p < 0.05, **p < 0.01 and ***p < 0.001 vs. the model group, NS, not significant

As previously reported, *trex1* is a non-restriction nucleic acid exonuclease present in the cytoplasm that efficiently hydrolyzes excess DNA fragments in cells [34]. After the *trex1* gene is deleted, DNA fragments continue to build up in the body and activate the cGAS-STING pathway. This leads to more interferon and inflammatory factors being released, which is a major factor in the development of autoimmune diseases [35]. *trex1*-deficient mice also die one after another at week eight due to the development of multi-organ inflammation. Wild-type mice were used as controls. After TTN was given for 14 consecutive days, a variety of tissues were collected from the mice, in which pathological tissue changes and gene transcription levels were examined. In vivo, H&E staining revealed that TTN significantly reduced the infiltration of inflammatory factors in cardiac, hepatic, pulmonary, and renal tissues induced by the deletion of the *trex1* gene in mice, and at the same time, TTN had no other direct effects on mice (Fig. 5A). We also examined the expression of the same gene in different tissues of WT or *trex1*^*−/−*^ mice, and the results of the study show that that TTN was also effective in suppressing the expression level of type I interferon, interferon stimulated genes and inflammatory cytokines at the gene level as well (Fig. 5B–E). This suggests that TTN can effectively alleviate the autoimmune response in *trex1*^*−/−*^ mice.

### The therapeutic effect of TTN on LPS/D-GaIN-induced acute liver injury

**Fig. 6:**
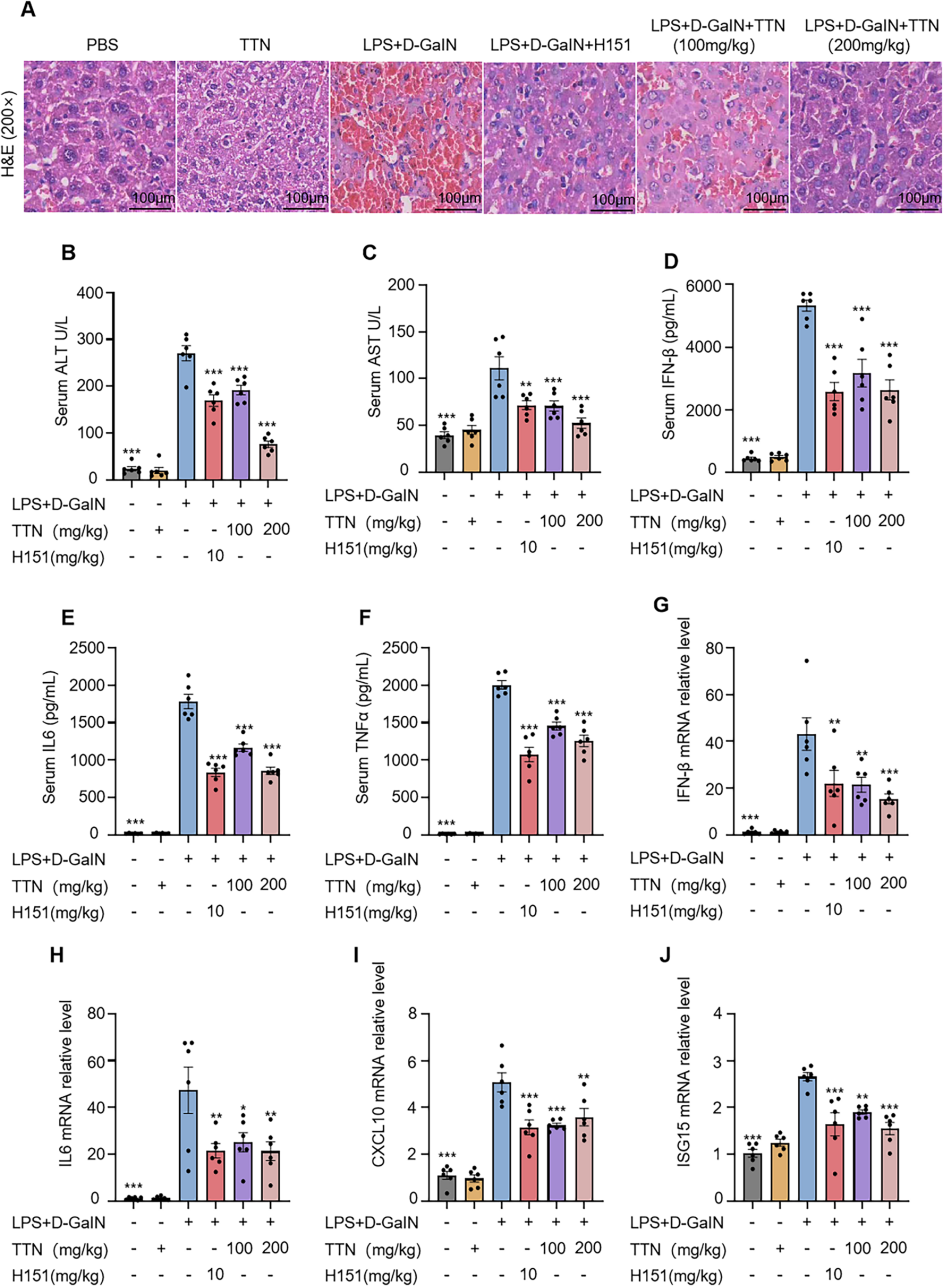
Therapeutic effect of TTN on LPS/D-GaIN-induced acute liver injury. C57BL/6 mice were divided into 6 groups (n = 6 per group), the administered group was gavaged with the corresponding dose of TTN for 7 consecutive days, and the remaining groups were given the excipients in the same manner. At the same time of the last administration, mice in the H-151 group were injected intraperitoneally with H-151 (10 mg/kg), and one hour later, except for the blank and TTN groups (200 mg/kg), the rest of the mice were injected intraperitoneally with LPS/D-GalN (2.5 mg/kg LPS and 250 mg/kg D-GalN). The samples were taken four hours later. **A** Representative images of H&E staining in liver tissues (n = 6 per group). **B**–**F** Mice were modeled by intraperitoneal injection of LPS/D-GaIN one hour after administration of vehicle or TTN, and the expression of ALT (**B**). AST (**C**), IFN-β (**D**), IL-6 (**E**), and TNF-α (**F**) in serum was measured four hours later (n = 6 per group). **G**–**J** Total RNA was extracted from mouse liver tissues by homogenization, and the transcript levels of IFN-β (**G**), IL-6 (**H**), CXCL10 (**I**) and ISG15 (**J**) genes were detected by qPCR assay(n = 6 per group). Data in (**B**–**J**) are presented as Mean ± SEM for the 6 mice per group, one-way ANOVA followed by Dunnett’s post hoc test was used to detect statistical differences between the analyzed multiple groups. *p < 0.05, **p < 0.01 and ***p < 0.001 vs. the model group, NS, not significant

We established the model of LPS/D-GaIN-medicated ALI in mice for evaluating the protective effect of TTN. LPS is an immunostimulant in the bacterial cell wall that elicits a strong immune-inflammatory response, which is enhanced by the addition of D-galactosamine in the liver [36]. It has been shown that under the influence of LPS/D-GaIN, the morphology of liver tissue undergoes major changes, with increased inflammatory infiltration, increased inflammatory factors, and many haemorrhages and injuries. In contrast, after administration of TTN or H151, which is a potent STING antagonist with significant inhibitory activity, mice underwent significant improvement in liver histomorphometry, with clear visual fields and a reduction in inflammatory factors and haemorrhage (Fig. 6A). We also tested liver function indices ALT and AST in mice by collecting serum and showed that the combination of LPS and D-GaIN impaired liver function in mice, but TTN or H151 reversed this lesion and lowered aminotransferase levels (Fig. 6B, C). Furthermore, we also detected changes of interferon and inflammatory factors in mouse serum by ELISA kits, and the results showed that TTN or H151 effectively decreased the elevation of interferon and inflammatory factors in mice (Fig. 6D–F). To further investigate whether TTN exerts therapeutic effects by affecting the cGAS-STING pathway, we analyzed the expression levels of type I interferon-related genes and inflammatory cytokines in liver tissues before and after TTN treatment, and the findings demonstrated that the reverse transcription level of IFN-β, IL6, CXCL10, ISG15 was markedly reduced by administration of either TTN or H151 compared with the model group (Fig. 6G–I). These results indicated that TTN may be able to improve ALI by regulating aberrant cGAS-STING pathway activation.

## Discussion

The cGAS in the cytoplasm recognizes DNA fragments of various sizes and is key to the body’s ability to fight pathogen infection, DNA damage and mitochondrial stress responses [37, 38]. The activated cGAS-STING pathway contributes to elevated levels of interferon and inflammatory factor expression. As a result, the cGAS-STING pathway is a crucial mediator between illness and the body's natural defenses and is involved in both preventing and treating disease [39, 40]. However, excessive release of interferon and inflammatory factors due to over-activation of the cGAS-STING pathway is an important cause of the development of related inflammatory and autoimmune diseases [41]. Targeted regulation of the cGAS-STING pathway to stabilize cytokines at normal levels is an important direction for autoimmune disease treatment and prevention [13]. Immunosuppressants and glucocorticoids are frequently used in clinical practice to treat inflammatory and autoimmune diseases, and despite their efficacy, they are associated with various side effects [42]. Therefore, the development of safe and effective drugs with few side effects is essential for the treatment of the disease.

Salvia miltiorrhiza is one of the widely used Chinese medicines for activating blood circulation and removing blood stasis, which is mainly used in the treatment of cardiovascular diseases and inflammatory diseases. At present, many Salvia miltiorrhiza preparations are widely used in clinical practice and have achieved ideal therapeutic effects. These preparations include Tanshinone capsules, Salvia miltiorrhiza granules, and Compound Dangshen Dropping Pills [43]. Tanshinones and salvianolic acid are the primary active ingredients in Salvia miltiorrhiza, with tanshinones being the primary liposoluble component [44]. Research has demonstrated that tanshinones possess anti-inflammatory and anti-tumor properties. For example, tanshinones can protect against acute lung injury through the PLCγ2/NLRP3 inflammasome signaling pathway [45]. Traditional Chinese medicines are characterized by multi-component and multi-target actions. The traditional preparations of Salvia miltiorrhiza commonly used in clinical practice usually consist of water-soluble or fat-soluble Salvia miltiorrhiza components. Consequently, we investigated the effect of TTN, a lipid-soluble fraction of Salvia miltiorrhiza, on the cGAS-STING pathway and its underlying mechanisms.

According to our findings, TTN can, in a dose-dependent manner, stop the aberrant activation of the cGAS-STING pathway that ISD causes in BMDMs and THP1 cells, which reduces the phosphorylation levels of STING and IRF3. Similarly, TTN also suppressed the amounts of IFN-β, IL6, TNFα, CXCL10, and ISG15 mRNA expression following abnormal activation of cGAS-STING signaling pathway. To further validate the effect of TTN on the cGAS-STING signaling pathway, we observed the effect of TTN using a model of cGAS-STING signaling pathway activation induced by various STING agonists (2',3'-cGAMP、diABZI、DMXAA). Consistent with the above findings, in the cGAS-STING pathway, which is triggered by several STING agonists, TTN reduced the phosphorylation of STING and IRF3, which in turn reduces the expression levels of inflammatory factors and type I interferon. When the RIG-I-MAVS pathway is activated, it can also raise the level of IRF3 phosphorylation. This causes type I interferon to be made. Consequently, we are also interested in learning whether TTN may have an effect on this pathway. After pretreatment of BMDM and THP1 cells with TTN, we modeled activation of the RIG-I-MAVS pathway using Poly(I:C) to find out what effect it has on this pathway. The results show that TTN can specifically inhibit ISD-induced IFN-β expression and Phosphorylation level of IRF3, however there was no inhibitory effect on IFN-β expression and Phosphorylation level of IRF3 induced by Poly(I:C) stimulation. Therefore, TTN is a compound that selectively and significantly inhibits DNA-activated immune pathways. We are still researching the precise mechanism of action at the cellular level in order to better understand the target proteins of TTN action. When 2′3'-cGAMP turns on STING, it oligomerizes and moves from the endoplasmic reticulum (ER) to the perinuclear Golgi intercompartment. The oligomerization of STING is necessary for the activation of TBK1 and the phosphorylation of STING. Therefore, we looked at TTN's function in the STING oligomerization process. The results indicate that TTN has no impact on the oligomerization of STING. 2',3'-cGAMP can bind to STING on the ER as the pathway's second messenger. STING recruits and activates TBK1, which in turn activates IRF3, forming the STING-TBK1-IRF3 signaling axis and causing downstream signaling. During this signaling process, activated TBK1 also, in turn, promotes the phosphorylation of STING. Phosphorylated STING binds to a positively charged region of IRF3, drawing IRF3 for phosphorylation by TBKl. An essential stage in the production of IFN-β and other immune factors is the interaction between STING, TBK1, and IRF3. Previous studies have demonstrated that overexpression of STING, TBK1, or IRF3 can induce upregulation of IFN-β expression. After pre-treatment with TTN, we separately transfect each of the above tagged proteins in HEK293 cells. IFN-β expression was measured by qPCR, and the findings indicated that the increase in expression level of IFN-β caused by transfection of these three tagged proteins could be inhibited by TTN. Therefore, based on the locations of STING, TBK1, and IRF3 in the cGAS-STING pathway, we hypothesized that TTN functions downstream of STING. To further elucidate the site of action of TTN in inhibiting the cGAS-STING signaling pathway, we conducted immunoprecipitation. The results showed that TTN did not affect the binding of STING-TBK1 and TBK1-IRF3, but attenuated the STING-IRF3 interaction, thereby inhibiting downstream signaling. This further suggests a potential mechanism for TTN to inhibit the cGAS-STING pathway, but the specific binding site needs to be further investigated.

To determine whether TTN can prevent the cGAS-STING pathway from being activated in vivo, We modelled the aberrant activation of cGAS-STING in vivo to further explore the effects of TTN. As expected, TTN greatly decreased the levels of interferon and inflammatory factors when we simulated DMXAA-induced STING activation in mice. This shows that TTN can also prevent the aberrant activation of STING and its downstream signaling pathways that are caused by STING agonists in vivo. The continuous activation of aberrant DNA for the cGAS-STING pathway is a major factor in the interferon buildup observed in *trex1*^*−/−*^ mice. Studies have shown that *trex1*^*−/−*^ mice's inflammation and mortality can be prevented by *cGAS*^*−/−*^ or *STING*^*−/−*^ regulation [46]. Autoimmune disorders linked to *trex1* deficiency are mediated by the cGAS-STING signaling pathway. Mice lacking *trex1* become ideal models for autoimmune disorders. According to recent genetic research, it is possible to prevent the aberrant buildup of DNA and the autoimmune system's overactivity by blocking the cGAS-STING signaling pathway at the gene level. These findings imply that the creation of inhibitors that target the cGAS-STING signaling pathway will have significant effects on the management of autoimmune disorders caused by *trex1*^*−/−*^*.* Thus, we investigated the possibility that TTN could be therapeutic for autoimmune disorders. The results showed that after 14 days of continuous administration of TTN, the inflammation level of *trex1*^*−/−*^ mouse tissues (heart, liver, lung, and kidney) were significantly reduced, and the mRNA levels of type I interferon, interferon stimulated genes and inflammatory cytokines in four tissues of *trex1*^*−/−*^ mice were also significantly inhibited. It is suggested that TTN has a good therapeutic effect on cGAS-STING pathway-mediated autoimmune diseases.

The liver, an essential metabolic organ, plays a crucial role in the body's metabolism of carbohydrates, proteins, and fats, as well as detoxification [47]. However, due to viral infections and improper medication use, the incidence of ALI is gradually increasing, and some patients even develop complications such as liver failure, hepatic encephalopathy, and hepatopulmonary syndrome [48]. Therefore, how to effectively prevent and treat ALI has become a research hotspot all over the world. Massive hepatocyte necrosis can be caused by D-GalN, and LPS can imitate the body's immune response after massive hepatocyte death. The ALI model induced by LPS combined with D-GalN is more in line with clinical practice. Research findings already show that combining D-GalN with LPS to create an ALI model triggers the activation of the cGAS-STING signaling pathway and During ALI, the organism's cellular damage or infection also triggers the activation of the cGAS-STING pathway [49]. H-151 lowers TBK1 phosphorylation and stops STING palmitoylation. This makes it a strong, selective, and covalent STING antagonist that works well in both vitro and vivo. Therefore, we used H151 as a positive control to evaluate the therapeutic effect of TTN after abnormal activation of the cGAS-STING pathway in vivo [50]. The study found that the liver tissue of mice in the D-GalN/LPS group had nuclear contraction, heavy congestion, and a lot of inflammatory cells. With the increase of dose concentration, the liver tissue of the TTN treatment group gradually returned to normal, while congestion and inflammatory infiltration decreased. ALT and AST elevations are significant indicators of liver damage. Following TTN treatment, the TTN treatment group's levels of ALT and AST were much lower than the model group’s, and the TTN group also had significantly lower serum levels of inflammatory factors and interferon such as TNFα, IL-6, and IFN-β. TTN also decreased the mRNA levels of inflammatory factors, type I interferon and interferon stimulated genes in liver tissues, according to the qPCR results. This implies that TTN may block the cGAS-STING pathway in order to fulfill its therapeutic function. However, high concentrations of TTN and H151 have similar therapeutic effects. This suggests that TTN’s role in other metabolic or non-metabolic pathways in living things needs to be studied further.

TTN, which possesses antibacterial and anti-inflammatory properties, is the major ingredient in tanshinone capsules. People frequently use it to treat a variety of acute and subacute inflammations, including tonsillitis and otitis externa, due to its good therapeutic impact. In July 2020, the State Drug Administration authorized tanshinone capsules (approval number Z13020110) for Sinopod. Tanshinone capsules have a significant anti-inflammatory effects, but their broad applicability is severely limited because the molecular mechanism behind this action is rarely described and the material basis for its efficacy remains unclear. On this basis, our study reported that TTN can play an anti-inflammatory role by inhibiting the cGAS-STING pathway and also provided a new idea for the treatment of autoimmune diseases and ALI by tanshinone capsule, which is conducive to its wider clinical application.

## Conclusion

TTN regulates abnormal cGAS-STING signalling pathway activation by modulating the interaction between STING and IRF3, demonstrating significant therapeutic potential for autoimmune diseases and ALI mediated by the cGAS-STING pathway.

## Supplementary Information

Supplementary Material 1.

## Data Availability

Data will be made available on request.
