# Correction: Total tanshinones ameliorates cGAS-STING-mediated inflammatory and autoimmune diseases by affecting STING-IRF3 binding

**DOI:** 10.1186/s13020-024-00996-w

**Published:** 2024-10-23

**Authors:** Chengwei Li, Jincai Wen, Xiaoyan Zhan, Wei Shi, Xiu Ye, Qing Yao, Simin Chen, Congyang Zheng, Xianlin Wang, Xinru Wen, Xiaohe Xiao, Yinghao Wang, Zhaofang Bai

**Affiliations:** 1 School of Pharmacy, Fujian University of Traditional Chinese Medicine, Fuzhou, China; 2 Department of Hepatology, The Fifth Medical Center of PLA General Hospital, Beijing, 100039 China; 3 Fifth Medical Center of Chinese, China Military Institute of Chinese Materia, PLA General Hospital, Beijing, China; 4 State Key Laboratory for Quality Ensurance and Sustainable Use of Dao-Di Herbs, Beijing, 100700 People’s Republic of China

**Correction to: Chinese Medicine (2024) 19:107** 10.1186/s13020-024-00980-4

Following publication of the original article [1], the authors reported that the vertical axis of Fig. 4D should be marked as "IFN-β", but it is mistakenly marked as "IL6". To ensure that readers receive accurate information, Fig. 4 has been updated and the authors declare that this correction does not affect the conclusion of the article.

The correct Fig. 4 has been provided in this Correction.

The incorrect Fig. 4 is:

**Fig. 4:**
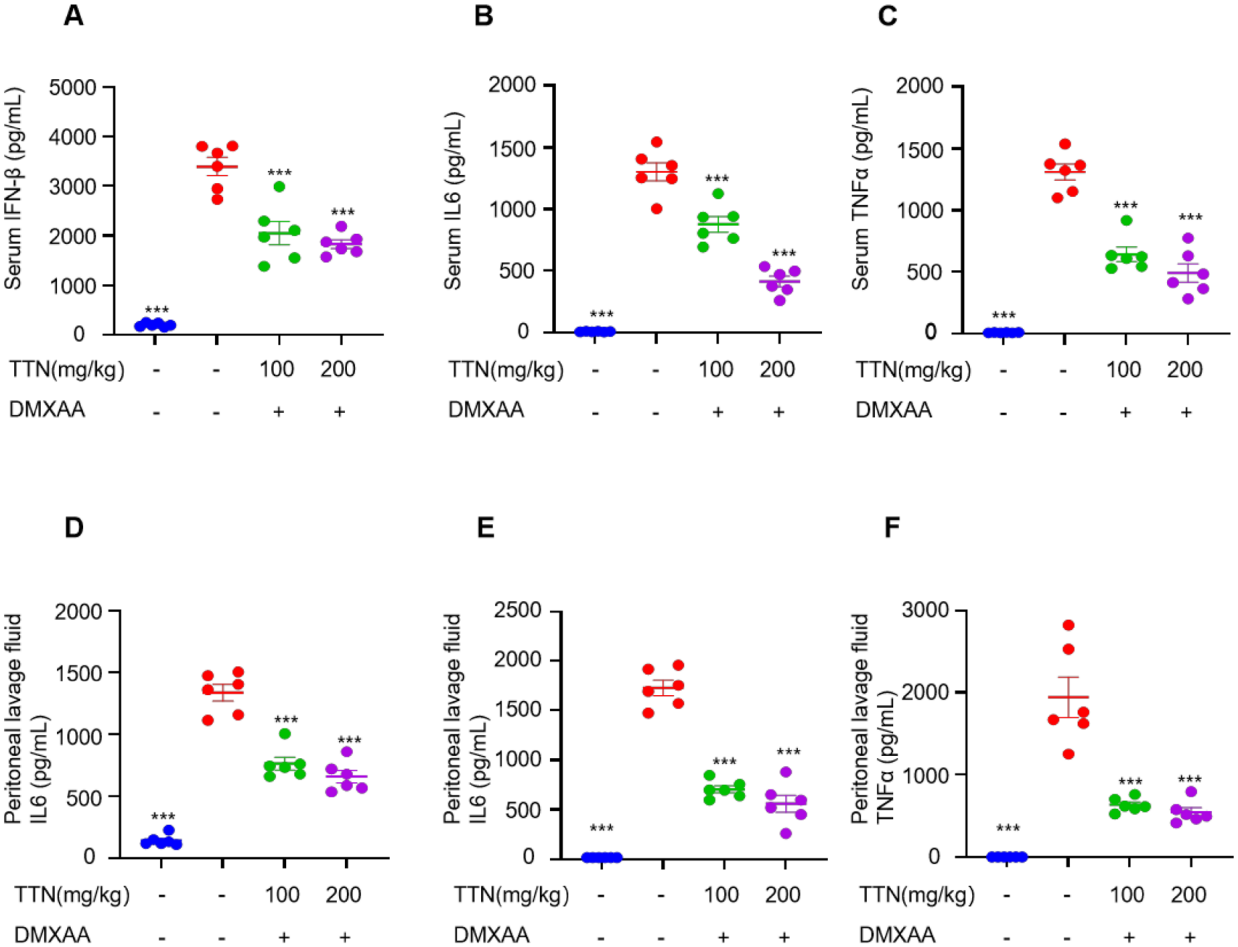
TTN inhibits DMXAA-induced activation of STING downstream signalling in vivo. **A–F** C57BL/6 J mice were randomly divided into four groups (n = 6 per group). The administration group received the appropriate dose of tanshinone by gavage for 7 consecutive days, while the blank and DMXAA groups received equal amounts of excipients, and one hour after the last administration, the mice received an intraperitoneal injection of DMXAA (25 mg/kg), except for the blank group. Serum and peritoneal lavage fluid were taken after four hours. The levels of IFN-β, TNF-α and IL-6 in serum and peritoneal lavage fluid were determined by enzyme-linked immunosorbent assay (n = 6 per group). Data in (**A–F**) are presented as Mean ± SEM, one-way ANOVA followed by Dunnett’s post hoc test was used to detect statistical differences between the analyzed multiple groups. *p < 0.05, **p < 0.01 and ***p < 0.001 vs. the model group, NS, not significant

The correct Fig. 4 is:

**Fig. 4:**
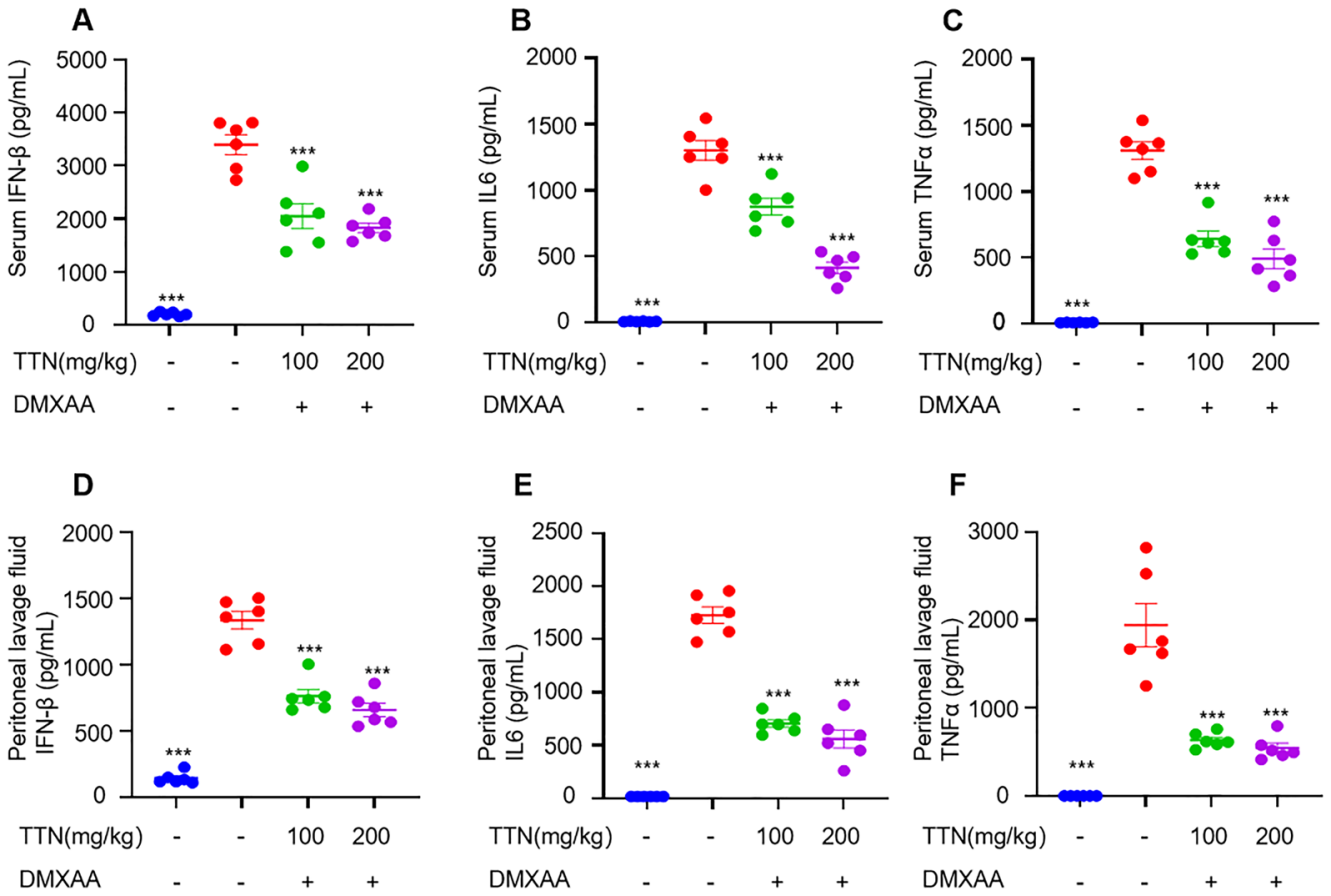
TTN inhibits DMXAA-induced activation of STING downstream signalling in vivo. **A–F** C57BL/6 J mice were randomly divided into four groups (n = 6 per group). The administration group received the appropriate dose of tanshinone by gavage for 7 consecutive days, while the blank and DMXAA groups received equal amounts of excipients, and one hour after the last administration, the mice received an intraperitoneal injection of DMXAA (25 mg/kg), except for the blank group. Serum and peritoneal lavage fluid were taken after four hours. The levels of IFN-β, TNF-α and IL-6 in serum and peritoneal lavage fluid were determined by enzyme-linked immunosorbent assay (n = 6 per group). Data in (**A–F**) are presented as Mean ± SEM, one-way ANOVA followed by Dunnett’s post hoc test was used to detect statistical differences between the analyzed multiple groups. *p < 0.05, **p < 0.01 and ***p < 0.001 vs. the model group, NS, not significant

The original article [1] has been corrected.
