# Supplementary material for: Total tanshinones ameliorates cGAS-STING-mediated inflammatory and autoimmune diseases by affecting STING-IRF3 binding
Source: Chin Med. 2024 Aug 15;19:107. doi: 10.1186/s13020-024-00980-4 (PMC11325629; doi:10.1186/s13020-024-00980-4)
Supplement: Supplementary file 1 — Supplementary Material 1. [file 13020_2024_980_MOESM1_ESM.docx]

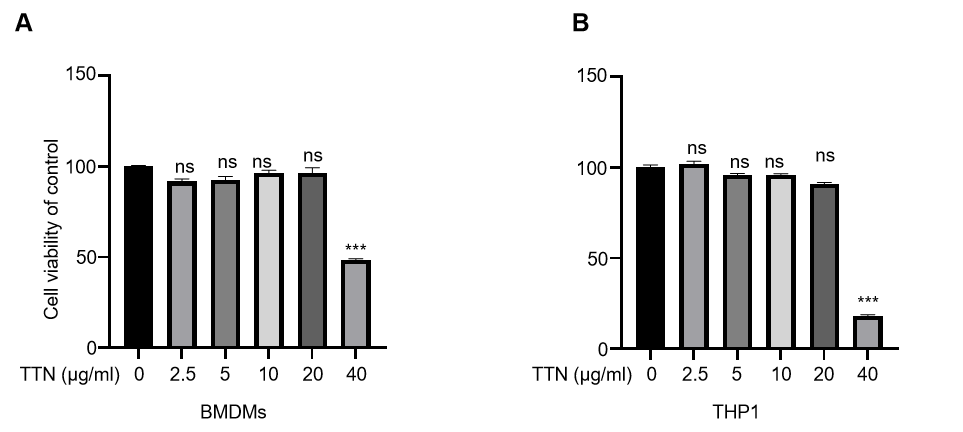


**Supplementary Figure 1 Cell viability testing of TTN in BMDMs and THP-1**

(A) The effect of different concentrations of TTN in BMDMs cell viability was detected by CCK8 toxicometry.

(B) The effect of different concentrations of TTN in THP-1 cell viability was detected by CCK8 toxicometry.

Data in (A-B) are presented as Mean ± SEM, one-way ANOVA followed by Dunnett’s post hoc test was used to detect statistical differences between the analyzed multiple groups. *p < 0.05, **p < 0.01 and ***p < 0.001 vs. control group, NS, not significant.


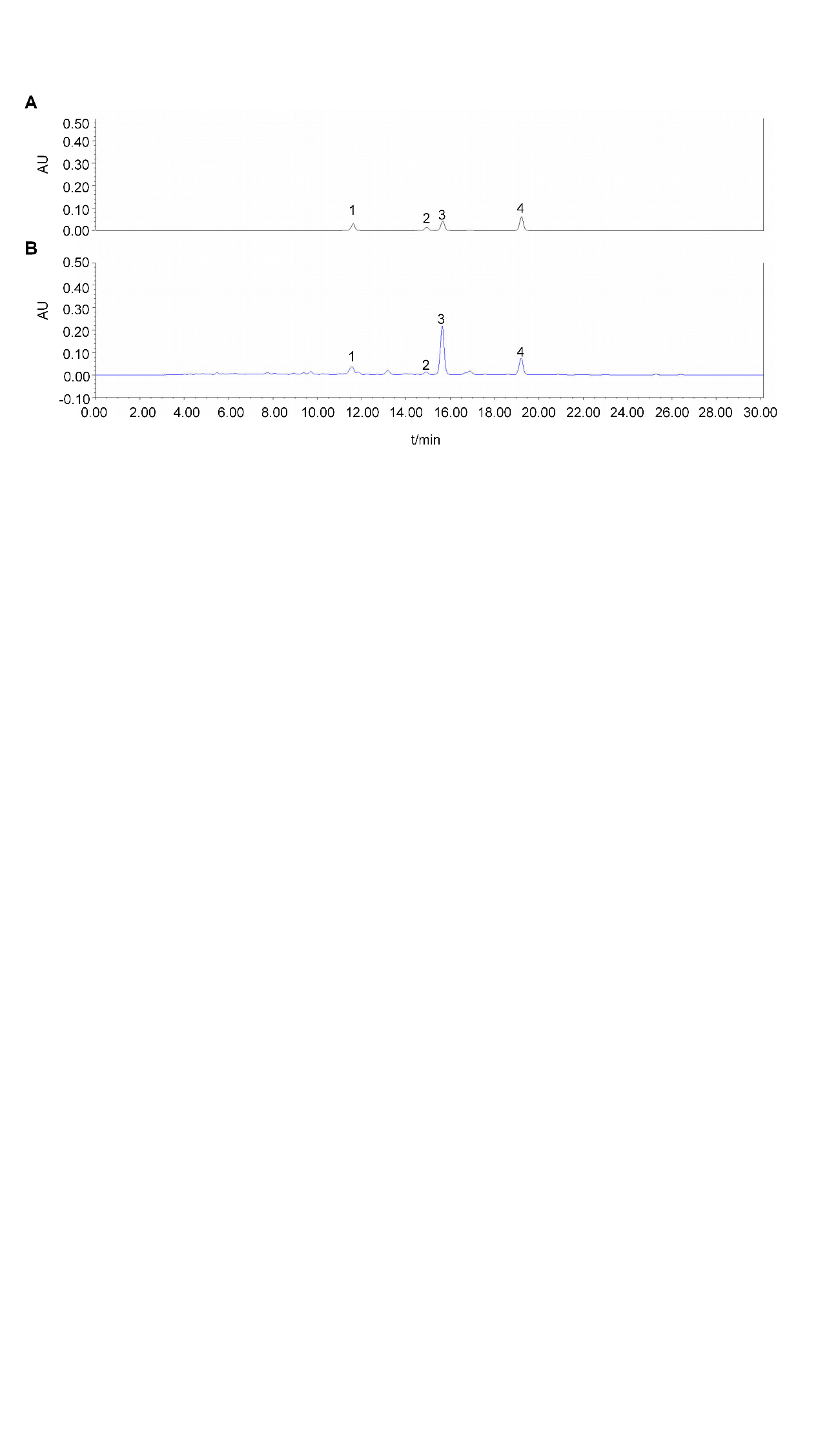


**Supplementary Figure 2 Quality control of TTN**

UHPLC analysis of the TTN. Mobile phase: methanol as mobile phase A, 0.1% phosphoric acid solution as mobile phase B; Detection wavelength: 270nm; Column temperature: 30℃; Flow rate: 0.8ml/min. (A) Mixed standard of a reference substance. (B) TTN fingerprint. Peak 1: Dihydrotanshinone I; Peak 2: Tanshinone I; Peak 3: Cryptotanshinone; Peak 4:Tanshinone IIA.
